# Genome-Wide SNP Analysis Reveals the Unique Genetic Diversity Represented by Fat-Tailed Coarse-Wooled Sheep Breeds of Kazakhstan

**DOI:** 10.3390/biology14111478

**Published:** 2025-10-23

**Authors:** Kairat Dossybayev, Makpal Amandykova, Daniya Ualiyeva, Tilek Kapassuly, Altynay Kozhakhmet, Elena Ciani, Bakytzhan Bekmanov, Rauan Amzeyev

**Affiliations:** 1 Laboratory of Animal Genetics and Cytogenetics, RSE Institute of Genetics and Physiology, Committee of Science of the Ministry of Science and Higher Education of the Republic of Kazakhstan, 93 Al-Farabi Avenue, Almaty 050060, Kazakhstan; kairat1987_11@mail.ru (K.D.); daniya.2010@mail.ru (D.U.); tilek.kapas@mail.ru (T.K.); altynaitg@gmail.com (A.K.); bobekman@rambler.ru (B.B.); 2 Laboratory of Molecular Genetic Examination, LLP «Kazakh Research Institute of Animal Husbandry and Fodder Production», 51 Zhandosov Street, Almaty 050071, Kazakhstan; 3 Faculty of Biology and Biotechnology, Al-Farabi Kazakh National University, 71 Al-Farabi Avenue, Almaty 050040, Kazakhstan; 4 Laboratory of Ornithology and Herpetology, Institute of Zoology, Committee of Science of the Ministry of Science and Higher Education of the Republic of Kazakhstan, 93 Al-Farabi Avenue, Almaty 050060, Kazakhstan; 5 Chengdu Institute of Biology, Chinese Academy of Sciences, 23 Qunxian South Street, Chengdu 610041, China; 6 Department of Biosciences, Biotechnologies and Environment, University of Bari, 1 Piazza Umberto I, 70121 Bari, Italy; 7 Faculty of Natural Sciences and Geography, Abai Kazakh National Pedagogical University, 13 Dostyk Avenue, Almaty 050010, Kazakhstan; rauanbiolog@gmail.com

**Keywords:** *Ovis aries*, Kazakh fat-tailed coarse-wooled sheep breed, Edilbay sheep breed, Gissar sheep breed, genome-wide SNP genotyping, genetic structure, genetic diversity, phylogenetics

## Abstract

**Background:** The fat-tailed coarse-wooled sheep breeds exhibit excellent reproductive performance, exceptional adaptability to pasture conditions, and high precocity, contributing to enhanced meat, fat, and wool productivity in sheep breeding. Despite the significant role of these sheep breeds in Kazakhstan’s livestock production, their genetics remain poorly studied. This raises concerns about the potential loss of unique, breed-specific traits that could be important for the future development and resilience of Kazakh stan’s sheep farming sector. This study aimed to analyze genome-wide genotyping SNP data of local fat-tailed coarse-wooled sheep breeds (Kazakh fat-tailed coarse-wooled, Edilbay, and Gissar) to reveal their genetic diversity, breed characteristics, and phylogenetic relationships with worldwide domestic sheep breeds and wild sheep. **Methods:** The OvineSNP50 Genotyping BeadChip was used to obtain genome-wide SNP genotyping data from 160 fat-tailed coarse-wooled sheep from Kazakhstan. Population structure analysis, principal component analysis, phylogenetic and the maximum likelihood tree analysis were performed in comparison with foreign domestic sheep breeds and wild sheep populations. **Results:** Kazakh breeds exhibited high genetic diversity, with Edilbay showing the greatest allelic richness. PCA and Admixture revealed clear differentiation among the three breeds: Edilbay and Gissar formed homogeneous clusters, while Kazakh fat-tailed coarse-wooled sheep displayed admixture and substructure. Evidence of gene flow from Edilbay into other Kazakh populations supports its role as a genetic source for regional breeds. Phylogenetic analysis placed Kazakhstani sheep close to other Central Asian breeds, while clearly distinct from East Asian and European populations. Wild sheep (Argali and Urial) formed separate clades, with Kerman wild sheep clustering closer to Urial. **Conclusions:** Our results highlight the value of genotyping data for studying genetic diversity and population structure. Developing genetic resources for Kazakhstan’s native sheep breeds will help preserve their unique diversity and ensure it remains available for future use in breeding and adaptation efforts.

## Simple Summary

Sheep farming is an important part of agriculture in Kazakhstan, especially fat-tailed breeds that are well adapted to the local environment. Namely, Kazakh fat-tailed coarse-wooled, Edilbay, and Gissar sheep breeds are valued for their ability to thrive in harsh pasture conditions, their early maturity, and production of high-quality meat, fat, and wool. Despite their economic and cultural importance, little is known about the genetic background of these animals. In this study, we analyzed genome-wide SNP data from 160 animals representing the three main local breeds to gain insights into their diversity and relationship with other sheep worldwide. Our aim was to explore what makes these breeds unique and how they have developed over time. Understanding their genetics is essential for improving breeding programs, protecting valuable traits, and ensuring the long-term sustainability of sheep farming in Kazakhstan. It also contributes to preserving the country’s agricultural heritage and supporting food security in the region. Our findings show that the Edilbay breed possesses particularly high genetic diversity and forms a distinct group, while some gene flow was also detected among the studied populations. Overall, the native sheep of Kazakhstan represent a significant reservoir of genetic variation in Central Asia, underscoring their importance for conservation and future breeding strategies.

## 1. Introduction

Sheep breeding plays a significant role in the agricultural economy of Kazakhstan, contributing approximately 6% to the overall livestock production value [1], with the sheep population reaching around 19.4 million by 2024 [2]. The most important breeds in Kazakhstan’s sheep industry are the fat-tailed coarse-wooled sheep, which make up approximately 50–55% of the country’s total sheep population [3]. This group includes major breeds such as Edilbay, Kazakh fat-tailed coarse-wooled, and Gissar, as well as more recently developed derived breeds like Saryarka, Ordabasy, and Baisary created through targeted crossbreeding [3]. Fat-tailed coarse-wooled sheep are predominantly maintained for meat and fat production. Among them, Edilbay sheep is highly adapted to the harsh breeding environments of Kazakhstan’s dry steppe, semi-desert, and desert zones, and are recognized for their excellent slaughter yields and high meat quality [4,5]. The Kazakh fat-tailed coarse-wooled sheep breed, developed through folk selection, is widely distributed across nearly all meat-fat sheep breeding regions of Kazakhstan and is known for its endurance, adaptability, early maturity, and excellent meat-fat quality [6]. Additionally, the Gissar breed, the largest among all domesticated sheep breeds worldwide, is characterized by its remarkable resilience, distinctive body structure, and exceptionally large fat tail [6]. Originated in Tajikistan [7], the Gissar sheep was introduced to Kazakhstan, Uzbekistan and Kyrgyzstan during the Soviet era, where it was widely used as an improver breed for meat and fat production [8].

Despite the unique characteristics of Kazakh fat-tailed coarse-wooled sheep breeds, which have been used in selective breeding programs both nationally and internationally, their genetic background, structure, and diversity remain insufficiently characterized. Understanding the genetic background of Kazakhstan’s fat-tailed coarse-wooled sheep breeds is crucial for uncovering their unique adaptive traits, guiding effective conservation strategies, and preserving valuable genetic resources for future breeding improvements.

In general, domestic sheep (*Ovis aries*) are reported to have originated from the wild Asian mouflon (*Ovis orientalis*) [9], with domestication events dated between approximately 11,000 and 10,500 years before present [10]. Following domestication, sheep dispersed from Southwest Asia to other regions of the world [11]. Subsequent mitochondrial DNA (mtDNA) analyses have revealed that domestic sheep are derived from two distinct ancestral maternal sources, with no evidence of genetic contributions from wild species such as urial or argali sheep [12]. The Edilbay sheep breed was also included in the sudy of Hiendleder et al. (1998) [12], which highlighted its distinct phylogenetic position, as it clustered separately from other domestic breeds. This further increases the interest in conducting a more detailed investigation of the genetic origins and relationships of Kazakhstan’s fat-tailed breeds.

Recent advancements in chip array-based genotyping technologies have enabled the detection of thousands of genomic variations across a wide range of species [13,14,15], including sheep [16]. These technological advances have established genome-wide SNP genotyping as a highly informative and relatively efficient method for studying the genetic structure of animal populations.

Previous investigations into the genetic diversity and phylogenetic relationships of Kazakh sheep populations have utilized various approaches, including 12 microsatellite markers [17] and mitochondrial DNA D-loop sequences [18]. In a more recent study, Kazakh fat-tailed coarse-wooled sheep were analyzed using runs of homozygosity (ROH) data, which identified several candidate genes—such as *BMP2* and *CLOCK* associated with productive traits [19]. Additionally, genome-wide SNP genotyping was applied to characterize the genetic structure of the newly developed Baisary fat-tailed breed, revealing a unique genome composition distinct from its progenitor breeds [20]. In the Saryarka fat-tailed coarse-wooled breed SNP-based genome-wide association studies (GWAS) identified genes linked to meat productivity traits [21]. Although Kijas et al. conducted a genome-wide SNP analysis involving numerous sheep breeds from around the world [16,22], their study did not include Central Asian breeds. Another investigation based on Whole-Genome Resequencing data included some fine-wool Kazakh breeds and the Edilbay breed [23]; however, because it encompassed 158 diverse domestic populations, it did not provide a detailed characterization of the genetic background and structure of Kazakh sheep breeds.

Hence, while previous studies have provided valuable insights into the genetic characteristics of Kazakh sheep, many relied on limited molecular markers or were narrowly focused on specific traits such as meat productivity. Moreover, large-scale international analyses often excluded Central Asian breeds or included Kazakh breeds only in broad, multi-breed comparisons that lacked detailed resolution. These limitations underscore the need for a more comprehensive investigation into the genetic background and structure of Kazakhstan’s fat-tailed coarse-wooled sheep breeds. In the present study, we analyzed genome-wide SNP genotyping data from 160 animals representing three native Kazakh breeds to gain deeper insights into their genetic diversity and breed-specific features. Additionally, we performed comparative analyses with wild sheep and globally distributed domestic breeds to explore their phylogenetic relationships and better understand the genetic structure of these important regional populations.

## 2. Materials and Methods

### 2.1. Animal Sampling and Data Collection

**Figure 1:**
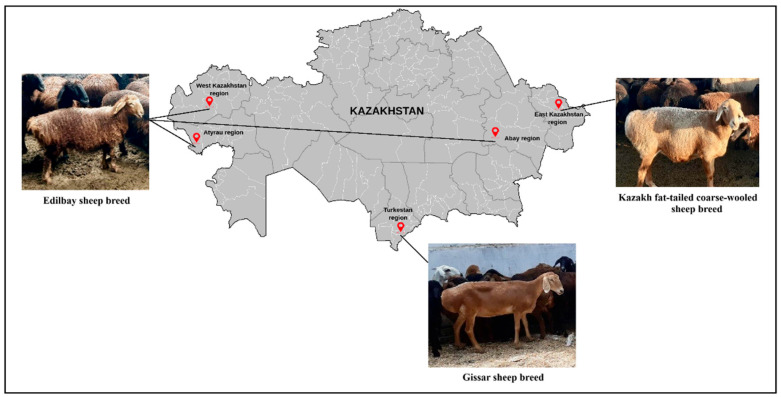
Geographic origins and sample localities of the studied sheep breeds from Kazakhstan (photographs were taken by the authors during farm visits for sample collection).

For this study, blood samples were obtained from 160 randomly selected 2–3 years old animals (Table 1) representing three sheep breeds—Edilbay (ED), Kazakh fat-tailed coarse-wooled (KFTCW), and Gissar (GISS)—raised in five farms across Kazakhstan (Figure 1) and according to FAO Animal Production and Health Guidelines (2011) [24].

Sheep under study were raised under traditional pastoral systems with predominantly natural mating. Blood samples from each animal were collected by an experienced veterinarian in accordance with all ethical standards and the animal study protocol approved by the Local Ethics Commission of the RSE Institute of Genetics and Physiology, Committee of Science, Ministry of Science and Higher Education of the Republic of Kazakhstan (Conclusion of the Local Ethics Commission No.6, 3 November 2022, Almaty, Kazakhstan). Blood samples were collected using EDTA vacuum tubes (Leuven, Belgium) and subsequently transported to the Laboratory of Molecular Genetic Examination of LLP “Kazakh Research Institute of Animal Husbandry and Fodder Production” for further analysis.

For the comparative genetic structure analysis of the studied sheep breeds with wild sheep representatives and worldwide domestic sheep breeds, SNP genotyping data from 11 both fat-tailed and thin-tailed domestic sheep breeds (across 8 countries and one geographic region) and three wild sheep (from seven countries) were obtained from the WIDDE sheep database [25], DRYAD [26], and Figshare [27] repositories (Supplementary Table S1). For the comparative genetic analysis, we first focused on fat-tailed sheep breeds from Central Asia. To assess the relatedness of Middle East breeds to those from Kazakhstan, we included breeds from Iran and Turkey. We then expanded the geographic scope of the study by adding sheep breeds from Europe and Africa, and finally incorporated one breed from Barbados and U.S. (BBB), which was previously reported by Kijas et al. (2012) [16] to have an admixed origin from African and European breeds, allowing us to test the reliability of our results.

### 2.2. DNA Isolation, Genome-Wide SNP Genotyping and SNP Data Filtering

Genomic DNA was extracted from sheep blood samples using the GeneJet Genomic DNA Purification Kit (Thermo Scientific, Waltham, MA, USA). DNA quality was assessed using NanoDrop One spectrophotometer (Thermo Scientific, Waltham, MA, USA) and quantified to a concentration of 50–100 ng/µL for subsequent SNP genotyping. SNP genotyping was performed using the OvineSNP50 Genotyping BeadChip, which includes 54,241 SNPs (Illumina, San Diego, CA, USA), on the iScan genotyping system (Illumina, San Diego, CA, USA).

The SNP datasets for all sheep included in this study were merged, and quality control filtering of SNPs was performed using PLINK 1.9 [28]. Only the SNPs common for all datasets were used for further population structure analysis. Several quality control parameters were applied to exclude low-quality SNPs, including marker missingness (--geno 0.05), individual missingness (--mind 0.05), Hardy–Weinberg equilibrium (--hwe hwe 1 × 10^−6^), and pruning of markers in approximate linkage equilibrium (--indep-pairwise 50 5 0.2).

### 2.3. Genetic Diversity and Phylogenetic Analyses

To estimate the genetic diversity levels in the studied domestic sheep populations the proportion of polymorphic SNPs (*Pn*), expected (*He*), and observed (*Ho*) heterozygosity and inbreeding coefficient (*F*) were observed using PLINK 1.9 [28] --het and --freq commands. Negative estimates usually arise from sampling variance or contrasting allele-frequency references [28,29] and were not further interpreted as biological signals. Allelic richness (*Ar*), which characterizes the overall genetic diversity and the presence of unique alleles within populations was calculated using rarefaction in ADZE 1.0 [30] to account for unequal sample sizes.

Phylogenetic relationships of three Kazakhstani sheep breeds with other domestic and wild sheep were determined using UPGMA method implemented in MEGA v.11 [31]. The visualization of the tree was performed in Figtree 1.4.4 [32].

### 2.4. Population Structure and Demographic Analyses

PLINK 1.9 [28] was used for principal components analysis and a graphical display of the PCA plots were generated using Python 3 [33]. Admixture 1.3 [34] was used to estimate genetic components of the studied populations and to determine their optimal number (*K*). The resulting plots were constructed on Clumpak [35].

Historical gene flow patterns among the studied domestic sheep breeds and wild sheep populations were depicted via the maximum likelihood tree-based approach implemented in TreeMix v.1.13 [36]. We removed SNPs with more than 5% missingness, then used the --freq option in PLINK to generate the required allele-count input. We tested models allowing 0 to 4 migration edges (−m 0 to −m 4), employing a block-jackknife of 500 SNPs (−k 500) to account for residual linkage. Argali was setted as an outgroup to root the tree, given its recognized genetic divergence of an extant wild representative toward domestic sheep. The model fit was assessed using residual plots generated by TreeMix, which display the residual covariance between observed and model-predicted allele frequencies. A well-fitting model exhibits minimal and symmetrically distributed residuals. The four-migration-edge model minimized these residuals and was selected accordingly.

Linkage disequilibrium (LD) decay was estimated using PLINK 1.9 [28], while effective population size (Ne) trajectories were inferred with SNeP 1.1 [37]. Before analysis, a more stringent LD pruning was applied (a 100-SNP window, step size of 50 SNPs, and *r*^2^ < 0.1). All plots were generated in R using the ggplot2 4.0.0 package [38].

## 3. Results

### 3.1. Genome-Wide SNP Genotyping Data Assessment

We generated novel genome-wide SNP genotyping data for 160 animals representing three fat-tailed coarse-wooled sheep breeds widely distributed across Kazakhstan. This dataset was used to investigate the genetic structure and phylogenetics of these populations in comparison with wild sheep and a range of global domestic sheep breeds.

SNP data analysis was conducted by dividing the dataset into three groups: (1) Kazakhstan sheep breeds only, (2) domestic sheep breeds only, and (3) all sheep under the study including domestic and wild sheep. Moreover, to obtain a clearer picture of the variation within domestic sheep breeds from Asia, we performed the PCA and Admixture including only Asian sheep breeds. For the analyses involving the domestic sheep groups and the combined group of all sheep (domestic and wild), only 20 animals from each Kazakhstan sheep breed (ED, KFTCW, and GISS) populations were randomly selected to balance the sample sizes across populations and to ensure the reliability of the analysis. A summary of the SNP quality control results is presented in Table 2.

Thus, the number of SNPs selected after the quality control assessment (column 7 of Table 2) was sufficient to conduct further analyses and ensure the reliability of the results obtained.

### 3.2. Genetic Diversity and Population Structure Analyses

Several parameters were calculated for each of the 14 domestic sheep populations in the study to evaluate within-breed genetic diversity. Wild sheep populations were excluded from this analysis due to their small sample sizes, which were insufficient to ensure reliable results (Table 3).

As shown in Table 3, the expected heterozygosity (*He*) varied from 0.316 in OUE-FRANCE to 0.384 in ED-KAZ, while the observed heterozygosity (*Ho*) ranged from 0.272 in OUE-FRANCE to 0.419 in NQA-NAMIBIA. The highest *He* values observed in ED-KAZ, KCW-KRG, GISS-KRG, QEZ-SW-ASIA and SOP-ITALY suggest relatively high genetic variability, whereas OUE-FRANCE and NQA-NAMIBIA showed reduced diversity. The proportion of polymorphic SNPs (*Pn*) was highest in ED-KAZ (98.90%), KFTCW-KAZ (98.54%), and GISS-KAZ (98.35%), indicating high levels of genetic diversity of sheep breeds from Kazakhstan. In contrast, non-Asian breeds such as OUE-FRANCE (79.97%) and NQA-NAMIBIA (74.21%) exhibited lower SNP polymorphism, suggesting reduced genetic variability or possibly historical bottlenecks or founder effects. Allelic richness (*Ar*) was highest in KCW-KRG (1.90427), QEZ-SW-ASIA (1.90433), and ED-KAZ (1.90702), reflecting a greater reservoir of genetic variation. In contrast, OUE-FRANCE (1.65567) and NQA-NAMIBIA (1.65979) exhibited notably lower allelic richness, consistent with their reduced levels of polymorphism and potentially indicating past demographic constraints or limited gene flow. The within-population inbreeding coefficient (*F_IS_*) values were generally low, ranging from –0.134 to 0.065, suggesting little or no inbreeding within most populations. Negative *F_IS_* values observed in Kazakh and Kyrgyz populations (ED-KAZ, KFTCW-KAZ, GISS-KAZ, and KCW-KRG) indicate a slight excess of heterozygotes, which may reflect balanced breeding strategies and large effective population sizes. In contrast, positive *F_IS_* values in OUE-FRANCE (0.154) and TIBQ-CHINA (0.065) suggest moderate inbreeding or substructuring effects within these populations. Pairwise population differentiation, expressed as mean *F_ST_*, ranged from 0.126 (ED-KAZ) to 0.301 (OUE-FRANCE), indicating moderate to high genetic differentiation among global sheep breeds. The lowest *F_ST_* values among Asian populations support their shared ancestry and relatively recent divergence compared to non-Asian groups.

**Figure 2:**
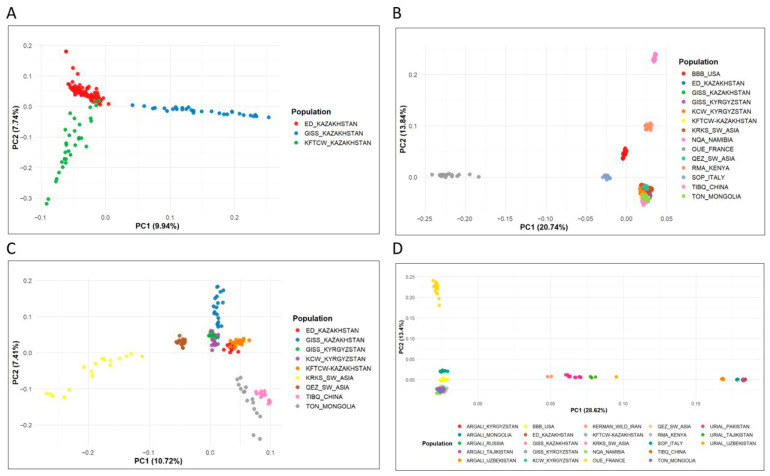
Principal Component Analysis (PCA) of the studied sheep populations. (**A**) PCA of the three studied sheep populations from Kazakhstan. (**B**) PCA of the studied global domestic sheep breeds. (**C**) PCA of the studied Asian domestic sheep breeds. (**D**) PCA of the studied wild sheep and global domestic sheep breeds.

Principal component analysis (PCA) was performed to examine the population structure of the studied sheep breeds (Figure 2).

As shown in Figure 2A, the ED-KAZ population forms a dense, compact cluster, indicating low intra-population variance. In contrast, the KFTCW-KAZ population displays a distinct vertical spread along PC2 on the left side of the plot, suggesting greater internal variability along this component and a clear separation from the other groups. The GISS-KAZ population is distributed horizontally along PC1 on the right side of the plot, indicating differentiation primarily along this axis. The PCA clearly distinguishes the three populations, revealing underlying genetic structure. The separation along the first two principal components highlights strong group-specific patterns of variation.

Subsequently, PCA was performed for worldwide domestic sheep populations (Figure 2B) and domestic sheep populations from Asia (Figure 2C). The first two principal components (PC1 and PC2) in Figure 2B reveal a clear separation of populations based on their geographical origin. Breeds from Europe (SOP-ITALY, OUE-FRANCE), Africa (NQA-NAMIBIA, RMA-KENYA), and from the Barbados and U.S. (BBB-USA) are well differentiated from Asian populations, reflecting strong genetic structure among the studied groups. African breeds, together with the BBB and SOP-ITALY populations, are distributed vertically along PC1 on the right side of the plot, whereas the OUE-FRANCE population is positioned distinctly on the left side along PC1, separate from the other populations.

Since all Asian sheep breeds clustered within a single region of the plot with substantial overlap in Figure 2B, a separate PCA was performed focusing exclusively on Asian breeds to explore their distribution in greater detail (Figure 2C). In this plot, TON-MONGOL and TIBQ-CHINA form two distinct and well-separated clusters, reflecting the unique genetic signatures of East Asian breeds compared with other Asian breeds in the study. Breeds from South-West Asia (KRKS-SW-ASIA and QEZ-SW-ASIA) also form distinct clusters. Populations from Kyrgyzstan (GISS-KRG and KCW-KRG) appear in proximity, suggesting relatedness with regional divergence. Interestingly, the Gissar breed from Kazakhstan (GISS-KAZ) is positioned far from the other sheep breeds from Kazakhstan (ED-KAZ and KFTCW-KAZ), but clusters closely with the Gissar breed from Kyrgyzstan (GISS-KRG). ED-KAZ and KFTCW-KAZ show clear genetic differentiation but cluster relatively close together, consistent with their shared geographic origin in Kazakhstan.

Finally, PCA was performed for all populations included in the study, encompassing both global wild sheep and domestic sheep breeds (Figure 2D). The PCA plot reveals clear genetic structuring among global sheep populations, including both wild and domestic sheep. The first principal component (PC1) primarily separates the wild Argali populations from all domestic breeds and Urial populations, indicating deep genetic divergence between the Argali lineage and domestic sheep. Meanwhile, Urial populations form a distinct cluster located between the Argali and domestic breeds, consistent with their phylogenetic status as a separate wild species. African sheep populations (NQA-NAMIBIA and RMA-KENYA) form clearly separated clusters along PC2, distinct from other domestic breeds, confirming their genetic differentiation and geographic origin from a separate continent. The BBB-USA breed is positioned between the Eurasian and African breeds, suggesting a potential admixture or intermediate genetic profile. Interestingly, European sheep breeds (SOP-ITALY and OUE-FRANCE) are genetically distinct and well-separated from Asian breeds, particularly along PC2, highlighting the presence of population-specific variation not captured by PC1 alone. Among the domestic populations, Asian breeds form compact, well-defined clusters, suggesting high within-group genetic homogeneity and marked between-group divergence, consistent with their shared geographic origin.

**Figure 3:**
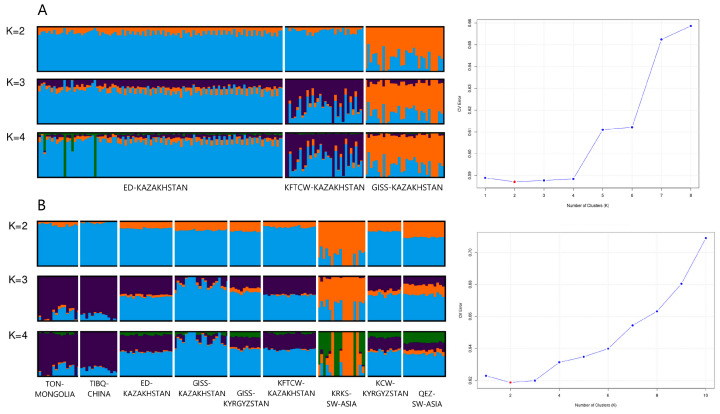
Admixture analysis of the studied sheep breed populations from Kazakhstan (**A**) (optimal *K* = 2) and of Asian domestic breeds (**B**) (optimal *K* = 2).

Next, Admixture analysis was performed to assess the genetic components of the sheep breeds from Kazakhstan and Asian domestic sheep breeds (Figure 3), as well as among the global domestic sheep breeds and wild sheep species (Supplementary Figure S1).

Admixture analysis shown in Figure 3A reveals the population structure of the three groups (ED-KAZ, KFTCW-KAZ, and GISS-KAZ) across *K* = 2 to *K* = 4 genomic components, with the lowest cross-validation error observed at *K* = 2. At *K* = 2, ED-KAZ and KFTCW-KAZ animals are predominantly assigned to a single genetic cluster (blue), while GISS-KAZ is largely represented by a distinct cluster (orange). At *K* = 3, a third cluster (purple) emerges, contributing mainly to KFTCW-KAZ, indicating internal substructure within this population. At *K* = 4, a fourth cluster (green) appears sporadically within ED-KAZ, though it does not constitute a major component in any group, potentially reflecting low-level admixture.

Admixture plot in Figure 3B illustrates the genetic components of Asian domestic sheep populations under varying assumptions of genetic components clusters. The Admixture results at *K* = 2 reveal a major division: KRKS-SW-ASIA is dominated by an orange cluster, while the remaining populations share a largely homogeneous blue cluster. At *K* = 3, a third (purple) cluster emerges, particularly prevalent in TON-MONGOL and TIBQ-CHINA, indicating deep genetic divergence between East Asian and Central/Western Asian breeds. At *K* = 4, a fourth cluster (green) appears, especially in KRKS-SW-ASIA, KCW-KRG, and QEZ-SW-ASIA, suggesting finer-scale differentiation among Central Asian and Middle Eastern populations. Admixture signals are evident in transitional populations such as KFTCW-KAZ and QEZ-SW-ASIA, reflecting gene flow or shared ancestral components.

Supplementary Figure S1A presents the Admixture results for global domestic breeds, including sheep breeds from Europe, Africa, and the Americas. Based on the results at the optimal value of *K* = 8, the domestic sheep populations exhibit distinct genetic clusters with varying degrees of admixture. East Asian populations such as TON-MONGOL and TIBQ-CHINA display relatively homogeneous genetic components, indicating limited recent gene flow from other groups. In contrast, Central Asian populations like ED-KAZ and GISS-KAZ show considerable admixture, suggesting historical or recent introgression from multiple genetic backgrounds. Notably, KRKS-SW-ASIA, OUE-FRANCE, NQA-NAMIBIA, RMA-KENYA, BBB-USA, and SOP-ITALY are characterized by unique and dominant ancestry components, pointing to their genetic divergence and possibly long-term isolated breeding.

The Admixture analysis of the global dataset, including both domestic and wild sheep populations, reveals clear genetic separation based on geographic origin (Supplementary Figure S1B). The optimal number of genetic components clusters was determined to be *K* = 8, based on the lowest cross-validation error. At *K* = 7, wild and domestic populations are well differentiated, with Argali and Urial sheep forming distinct genetic components. Domestic breeds exhibit a high degree of ancestry homogeneity, particularly among Asian populations, which are predominantly represented by a blue cluster. The KERMAN_WILD-IRAN population shows greater genetic affinity with Urial than with Argali, suggesting closer evolutionary relatedness. Overall, the population structure observed at *K* = 7 remained consistent at *K* = 8 and *K* = 9, without introducing substantial changes.

### 3.3. Phylogenetics and Demographic Analyses, and Breed Differentiation

**Figure 4:**
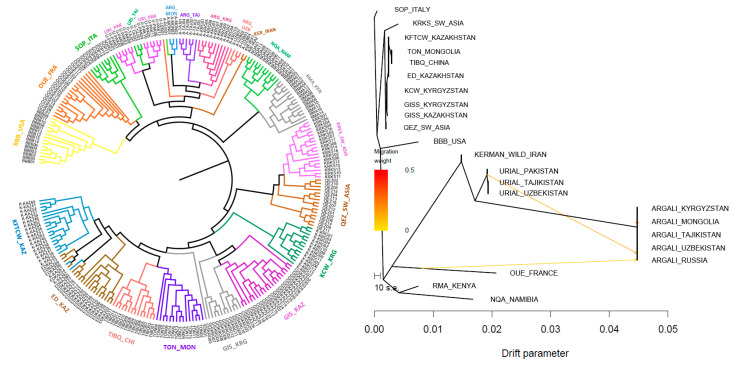
(**A**) Phylogenetic tree depicting relationships among the studied sheep populations. (**B**) TreeMix plot showing the maximum likelihood tree and inferred admixture events among the studied sheep populations.

To elucidate the genetic relationships among the studied wild and domestic sheep populations, an un-rooted neighbor-joining phylogenetic tree was constructed based on genome-wide SNP data (Figure 4A).

The circular phylogenetic tree displays distinct clustering patterns consistent with population identity and geographic origin. Animals from the same population clustered closely, reflecting strong within-group genetic similarity. The European sheep from France and Italy clustered together with the breed from USA reflecting the probable common ancestry or possible introgression and forming a monophyletic clade to the wild representatives from Pakistan, Mongolia and Central Asia. African sheep breeds from Namibia and Kenya clustered a separate branch sharing geographic proximity. The Middle Eastern breeds (Turkey and Iran) clustered into a monophyletic group that was closely related to Central Asian and Chinese breeds, respectively.

TreeMix analysis was performed to investigate the relationships and potential gene flow among wild and global domestic sheep populations. The resulting maximum likelihood tree (Figure 4B) reveals distinct clustering of major lineages. The most optimal value of *m*, demonstrating the highest reproducibility and consistency across replicates, was determined to be 3. The ARGALI wild sheep form a deeply divergent clade, well separated from both Urial and domestic breeds. The URIAL-PAKISTAN populations occupy a basal position relative to domestic sheep, indicating a closer genetic relationship to domesticated forms than ARGALI. The KERMAN_WILD-IRAN population shows a closer genetic affinity to domestic breeds than to other wild lineages. Asian domestic sheep populations cluster together with short branch lengths, reflecting recent common ancestry or limited differentiation. The close genetic relationship among Central Asian breeds, where the Kazakh (ED, KFTCW, GISS) and Kyrgyz (GISS, KCW) populations formed a distinct subcluster, reflecting their shared ancestry and genetic continuity across the region. More geographically isolated breeds, such as SOP-ITALY, BBB-USA, RMA-KENYA and NQA-NAMIBIA form peripheral branches.

**Figure 5:**
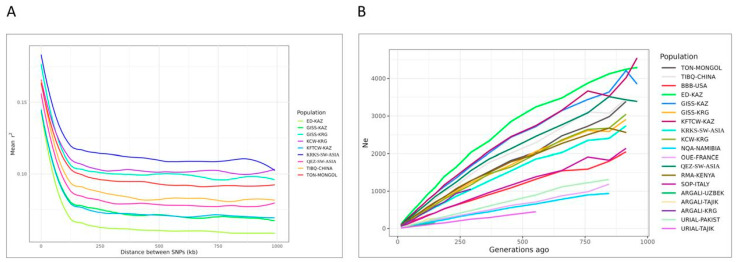
(**A**) Estimated linkage disequilibrium (LD) decay for Asian domestic sheep breeds under the study. (**B**) Estimated effective population size (Ne) for domestic sheep breeds and wild sheep under the study.

Next, we constructed linkage disequilibrium (LD) decay and effective population size (Ne) graphs (Figure 5A and Figure 5B, respectively) to assess patterns of genetic diversity and historical demography across populations and reveal recent population dynamics and long-term genetic structure. Summary statistics of pairwise LD (*r*^2^) values for each population are provided in Supplementary Table S2, while estimates of effective population size (Ne) obtained from LD-based analysis are summarized in Supplementary Table S3. These supplementary data complement the graphical results by presenting detailed numerical summaries for all analyzed sheep populations.

Wild sheep populations were excluded from the linkage disequilibrium (LD) decay analysis due to their small sample size, which was insufficient for reliable estimation of LD decay. Therefore, the LD decay graph shown in Figure 5A includes only Asian domestic sheep breeds, while Supplementary Figure S2 presents the LD decay graph for all sheep populations, including non-Asian domestic breeds and selected wild sheep populations with sufficient sample sizes for inclusion in the analysis.

The LD decay analysis (Figure 5A) illustrates the decline of linkage disequilibrium (measured as mean *r*^2^) with increasing physical distance between SNPs across multiple Asian domestic sheep populations. A typical LD decay pattern was observed, where *r*^2^ values decreased sharply within the first 100–200 kb and plateaued at greater distances. Among the populations, KRKS-SW-ASIA exhibited the slowest LD decay and the highest overall *r*^2^ values, suggesting extended linkage disequilibrium. In contrast, ED-KAZ showed the fastest LD decay and the lowest *r*^2^ values, indicating shorter haplotype blocks and potentially greater historical recombination. Populations such as GISS-KAZ, QEZ-SW-ASIA, and TON-MONGOL showed intermediate LD decay rates, reflecting varying levels of genetic diversity and effective recombination.

Figure 5B presents the estimated historical effective population sizes (Ne) for domestic and wild sheep populations over the past 100 generations. Domestic sheep from Kazakhstan (ED-KAZ, GISS-KAZ, and KFTCW-KAZ) exhibited relatively high Ne values with a steady increase in the distant past, reaching estimates between 3500 and 4000 animals. In contrast, wild sheep populations such as URIAL-PAKIST and URIAL-TAJIK demonstrated much lower Ne values, particularly in recent generations.

## 4. Discussion

In this study, we present the first systematic analysis of the genetic structure, phylogenetics relationships, and demographic history of three fat-tailed coarse-wooled sheep breeds from Kazakhstan in a global context. Our findings reveal high levels of SNP polymorphism and allelic richness, particularly the Edilbay breed, reflecting substantial genetic diversity likely maintained through large effective population size and extensive breeding history. The high genetic diversity observed in Edilbay sheep in our study is consistent with previous findings [17,20,39], which reported high levels of allelic richness and heterozygosity in this breed.

Population structure analyses (PCA and Admixture) revealed the well-defined clustering and minimal overlap among three Kazakh breeds, with Edilbay and Gissar forming genetically homogeneous groups and KFTCW-KAZ displaying higher internal variability. Evidence of Edilbay-specific components in Gissar and KFTCW-KAZ suggests historical introgression, possibly due to shared ancestry or past breeding practices. These results are consistent with our previous findings, which demonstrated clear genetic differentiation between the Edilbay and Gissar sheep breeds [20]. However, the admixture results also revealed traces of Edilbay-specific genetic components in the Gissar and KFTCW-KAZ breeds, suggesting that introgression from the Edilbay breed may have occurred historically. In a previous study comparing Kazakh sheep breeds with global sheep populations, Edilbay sheep were identified as direct descendants of historical domestic sheep ancestors [39]. Consistent with previous findings, our results suggest that the Edilbay breed may represent a genetic ancestral source for both the Kazakh fat-tailed coarse-wooled sheep and the Gissar breed. Additional support for this hypothesis comes from the mitochondrial DNA analysis conducted by Hiendleder et al. (1998) [12], which demonstrated that the Edilbay sheep diverged from wild sheep ancestors earlier than both the Gissar and Astrakhan breeds. This finding reinforces the notion that the Edilbay breed has ancient genetic roots and may predate several modern Central Asian breeds in terms of its evolutionary history. However, Deniskova et al. (2018) [40] proposed that the Edilbay breed originated from a cross between Kazakh fat-tailed coarse-wooled sheep and the Astrakhan breed. Nevertheless, this hypothesis is not yet confirmed by direct genetic evidence and as far as now no genetic data are available to support such hybrid origin. Furthermore, the available literature provides no concrete information regarding the precise origin, timeline, or breeding methods used in the development of the Edilbay sheep. In general, the breed is regarded as a product of traditional or folk selection practices carried out by local pastoral communities. It is also likely that the name “Edilbay” was derived from the name of a tribe or clan historically associated with the region where the breed was developed. On the contrary, the Astrakhan breed of sheep is considered to be descendant from the Edilbay breed. During the Great Jura of 1879, Kazakh nomads temporarily moved to the Astrakhan region in search of pastures. The descendants of Edilbay sheep left in this area over time formed an Astrakhan breed. Thus, the modern Astrakhan breed of sheep is in fact a genetic continuation of the Edilbay breed [41,42,43,44].

The PCA results highlight pronounced genetic structuring among global sheep populations, reflecting both their domestication history and geographic distribution. The clear separation of Argali and Urial from domestic breeds supports their deep evolutionary divergence and distinct phylogenetic status. Moreover, the genetic differentiation observed among African, European, and Asian domestic populations underscores the influence of geographic isolation and localized breeding histories on the genetic makeup of modern sheep breeds. Both PCA and Admixture revealed strong geographic clustering of global domestic sheep, with clear continental differentiation and fine-scale structure within Asia. Analyses highlighted the distinctiveness of East Asian breeds (e.g., TON-MONGOL, TIBQ-CHINA) and showed a close genetic relationship between GISS-KAZ and GISS-KRG. These results reinforce the hypothesis of historical genetic continuity and introgression across West, Central, and East Asian sheep lineages [23]. Also, these findings are in agreement with the results of Wei et al. (2015), who demonstrated by Admixture analysis that Kazakh sheep are genetically distinct from those of Tibet and China [45]. The strong genetic affinity observed between GISS-KAZ and GISS-KRG aligns with previous studies [21], suggesting that the Gissar breed originated from a single historical and geographical region—the Pamir-Alai mountainous region [46]. During the Soviet era, active exchange of livestock among Kyrgyzstan, Tajikistan, Uzbekistan, and Kazakhstan [47,48] likely facilitated gene flow and contributed to the preservation of genetic similarity across regions. Additionally, breeding programs in these countries often targeted similar traits (e.g., live weight, fat deposition in the rump), which may have reinforced a shared genetic structure. It is also important to note that the SNP panel used was of medium density and did not cover the entire genome, potentially limiting the detection of highly polymorphic regions or signals of selection. On the other hand, the development of the Ovine SNP50 BeadChip did not adequately account for fat-tailed sheep, resulting in their underrepresentation in the SNP Discovery panel [49].

Additionally, Admixture analysis detected substructure within South-West Asian populations (KRKS-SW-ASIA and QEZ-SW-ASIA) that was not resolved by PCA, indicating its greater sensitivity to ancestral admixture and gene flow. The results confirm a stable genetic boundary between Iranian and Turkish breeds, shaped by geography and selection, consistent with previous studies [50,51]. It is known that Admixture models are generally more effective than PCA in detecting fine-scale ancestry and recent gene flow [52,53].

Both PCA and Admixture analyses consistently revealed strong genetic structuring among global domestic and wild sheep populations, particularly along geographic lines. The separation of wild species (Argali and Urial) from domestic breeds supports deep evolutionary divergence, while the intermediate position of Urial sheep aligns with their phylogenetic placement. The homogeneity of Asian domestic breeds observed in PCA is mirrored in Admixture by the dominance of a shared genetic cluster, highlighting their cohesiveness. Admixture and TreeMix further revealed common ancestry between Central Asian and Iranian sheep, corroborating the findings of Moosanezhad Khabisi et al. [54]. This shared ancestry likely reflects historical events such as nomadic expansions, military invasions, and trade along the ancient Silk Road.

The phylogenetic tree offers a clear visualization of the genetic divergence and genetic relationships among sheep populations. The deep separation of Argali from both Urial and domestic breeds supports its role as an evolutionarily distinct lineage, corroborating findings from TreeMix and PCA analyses. Meanwhile, Urial populations, while genetically distinct, are positioned closer to domestic lineages in spite of the fact that there is no evidence for contributions to domestication from urials [12]. Kerman wild sheep was determined by both genome-wide SNP and mtDNA analysis. SNP analysis showed that the Kerman sheep is an intermediate species between *O. vignei* and *O. gimelini*, while according to mtDNA close to *O. vignei* [55]. In our study, the Kerman wild sheep’s intermediate placement between wild and domestic clusters may indicate a historical admixture or transitional role in early sheep domestication in the Iranian Plateau. The tight clustering of Central Asian domestic breeds indicates genetic continuity, likely due to shared ancestry and gene flow, supporting earlier reports of a common genetic background among fat-tailed coarse-wooled sheep along the Silk Road [54].

Distinct branches of African (RMA-KENYA, NQA-NAMIBIA), American (BBB-USA), and European (SOP-ITALY, OUE-FRANCE) breeds point to geographic isolation and local adaptation, in line with phylogenetic clades reported in previous studies [16,23]. Overall, the phylogenetic results corroborate PCA and Admixture analyses, highlighting the complex history of domestication, divergence, and regional differentiation in sheep.

The TreeMix results provide valuable insight into the domestication and migration history of sheep. The deep divergence of Argali from all other groups supports its role as a genetically distinct wild ancestor not directly involved in sheep domestication. In contrast, the Urial populations, especially those from Pakistan, appear more closely related to domestic breeds. The genetic cohesion observed among Central Asian breeds supports a scenario of regional expansion from a common ancestral stock, while the longer branches of peripheral breeds may reflect geographic isolation and genetic drift. The detected migration event involving RMA-KENYA, NQA-NAMIBIA and BBB-USA into Central Asian domestic sheep implies historical gene flow, possibly mediated by transregional livestock trade or pastoralist movements between Africa and Asia. This signal of admixture highlights the complex demographic history of domestic sheep, shaped not only by domestication and divergence but also by later episodes of cross-continental gene flow.

The low and mostly negative *F_IS_* values in Central Asian breeds suggest that these populations maintain healthy levels of genetic variation, likely due to their large population sizes and traditional breeding practices that promote genetic mixing. This pattern aligns with the observed high heterozygosity and allelic richness in Kazakh and Kyrgyz sheep. Conversely, the elevated *F_IS_* in European and East Asian breeds (e.g., OUE-FRANCE, TIBQ-CHINA) may reflect population subdivision or recent inbreeding. The moderate *F_ST_* values among Asian populations indicate ongoing gene flow and shared historical origins, whereas higher *F_ST_* in geographically distant populations such as OUE-FRANCE and NQA-NAMIBIA reflects stronger genetic differentiation driven by geographic isolation and breeding system differences. These findings collectively emphasize the genetic cohesion within Asian sheep and their clear divergence from other continental groups.

The LD decay patterns among Asian domestic sheep populations reflect significant variation in demographic history and breeding structure. In KRKS-SW-ASIA, slow LD decay with high *r*^2^ values indicates reduced recombination and small effective population size, likely due to bottlenecks, inbreeding, or recent selection, consistent with Eydivandi et al. [50]. In contrast, rapid LD decay and lower *r*^2^ values in ED-KAZ suggest higher genetic diversity, larger effective population size, and more recombination, in line with its high polymorphism and allelic richness indicating that this population may have experienced fewer demographic constraints or more gene flow over time. These results agree with Pozharsky’s estimates of LD decay in Kazakh sheep breeds [37] and with ROH analyses showing that the Edilbay breed retains broad genetic diversity, likely due to its native status and balanced breeding strategies aimed at preserving genetic variability [56].

The Ne estimates further support this interpretation. Kazakhstani breeds (ED-KAZ, KFTCW-KAZ and GISS-KAZ) maintained large effective population sizes, reflecting stable breeding histories and the long-term preservation of genetic diversity. This pattern likely results from less intensive artificial selection, broad breeding networks, and the socio-economic importance of meat-fat–tailed sheep, which comprise over 70% of Kazakhstan’s sheep population [42]. These sheep are deeply rooted in traditional Kazakh pastoralism due to their exceptional adaptability to the harsh environmental conditions of arid and semi-arid zones. Among them, the Edilbay breed is the most widespread and serves as a key genetic resource for improving meat-fat traits in other native breeds. Its large Ne is directly linked to population size and management structure; for example, the “Birlik” farm maintains 16,512 Edilbay sheep, organized into flocks of 500–600 animals that promote genetic exchange and reduce inbreeding [17]. The breed’s wide distribution across diverse environments and the persistence of traditional folk selection further safeguard its gene pool. Similar results were reported by Deniskova et al. (2018) [40], with Central Asian breeds showing higher Ne compared to global populations [8], likely due to mass breeding, adaptability, and cultural importance. In contrast, the reduced Ne values observed in URIAL-PAKIST and URIAL-TAJIK likely reflect recent demographic declines driven by poaching, habitat loss, and competition with domestic livestock. Such pressures have fragmented urial populations into small, isolated groups, consistent with their documented conservation concern in both Pakistan and Tajikistan [57,58].

To sum up, the results of this study provide valuable insights into the demographic and evolutionary history of sheep populations in Central Asia, highlighting both diversity and connectivity in the region’s livestock genetic resources.

## 5. Conclusions

In summary, this study provides the first comprehensive genomic assessment of fat-tailed coarse-wooled sheep from Kazakhstan in the context of wild and globally distributed domestic sheep populations. Our results demonstrate that Kazakh breeds, particularly the Edilbay, harbor exceptionally high levels of genetic diversity, maintained through large effective population sizes and extensive breeding histories. Population structure analyses consistently revealed well-defined clustering among Kazakh breeds, with Edilbay and Gissar forming homogeneous groups, while Kazakh fat-tailed coarse-wooled sheep exhibited greater variability and signatures of admixture. The Edilbay breed emerged as a key ancestral genetic resource for Central Asian sheep, with evidence of historical introgression into Gissar and other regional breeds.

Comparative analyses further highlighted strong geographic structuring of global sheep populations, with clear separation between continental groups and fine-scale differentiation among Asian lineages. The genetic continuity observed across Central Asian, Iranian, and Chinese breeds underscores the role of historical migrations and trade along the Silk Road in shaping sheep diversity. Moreover, wild sheep species (Argali and Urial) were confirmed as deeply divergent lineages, with Kerman wild sheep positioned as a distinct urial subspecies closely related to domestic lineages on the Iranian Plateau. Together, these findings underscore Kazakhstan’s fat-tailed sheep as an important reservoir of genetic diversity and a cornerstone of Central Asian pastoral heritage. They provide a valuable genomic framework for future conservation, breeding, and sustainable management programs aimed at preserving both the productivity and adaptability of these historically significant breeds.

## Figures and Tables

**Table 1:** Sheep breeds used for the study and their characteristics.

No.	Sheep Breed	Farm	Number of Animals	Overall
1	Edilbay (ED)	“Birlik Mal Zauyty” LLP, Birlik village, Zhangaly district, West Kazakhstan region	33	97
		LLP “Suyindik asyl tukymdy koy zauyty”, Suyindik village, Kurmangazy district, Atyrau region	32	
		Peasant farm “Aigul”, Donenbay village, Ayagoz district, Abay region	32	
2	Kazakh fat-tailed coarse-wooled (KFTCW)	Peasant farm “Kundyzdy”, Kundyzdy village, Ulken Naryn district, East Kazakhstan region	32	32
3	Gissar (GISS)	Peasant farm “Darbaza”, Darbaza village, Saryagash district, Turkestan region	31	31
Overall				160

**Table 2:** Summary of SNP quality control results for sheep groups under the study.

Studied Sheep Groups	Total Number of Animals	Total Number of SNPs	Number of Animals After QC	Number of SNPs After QC	Number of Animals After LD Pruning	Number of SNPs After LD Pruning
Kazakhstani sheep breeds	160	51,102	160	47,969	160	37,969
Domestic sheep breeds	225	39,273	182	36,798	182	30,230
Domestic sheep breeds and wild sheep	267	38,667	258	31,339	258	27,045

**Table 3:** Within-breed genetic diversity parameters for the studied domestic sheep populations.

No.	Population	N	*He*	*Ho*	*F*	*F_IS_*	Mean *F_ST_*	*Pn*, %	*Ar*
1	ED-KAZ	20	0.383901	0.396967	−0.034014	−0.008059360	0.126425371	98.90	1.90702
2	KFTCW-KAZ	20	0.379533	0.390952	−0.030186	−0.005761707	0.133995353	98.54	1.89864
3	GISS-KAZ	20	0.378127	0.394446	−0.043173	−0.016917877	0.135257999	98.35	1.89474
4	TON-MONGOL	15	0.376177	0.369286	0.018922	0.048046452	0.142830552	96.76	1.88441
5	TIBQ-CHINA	14	0.375390	0.362380	0.035135	0.065535223	0.146539513	96.21	1.88215
6	KCW-KRG	13	0.381521	0.405609	−0.063152	−0.023469213	0.136567911	97.62	1.90427
7	GISS-KRG	12	0.380844	0.399926	−0.049898	−0.008697962	0.133568616	97.60	1.90648
8	KRKS-SW-ASIA	18	0.372922	0.394711	−0.058287	−0.027327093	0.156338863	96.15	1.86975
9	QEZ-SW-ASIA	15	0.382087	0.391640	−0.025053	0.007706401	0.131929368	98.02	1.90433
10	SOP-ITALY	9	0.382399	0.402810	−0.053236	−0.002107075	0.158924142	96.21	1.65776
11	OUE-FRANCE	18	0.316522	0.271522	0.142368	0.154365564	0.301822277	79.97	1.65567
12	BBB-USA	15	0.362649	0.366821	−0.011258	0.019518782	0.191684706	93.19	1.83533
13	RMA-KENYA	15	0.352499	0.372089	−0.055720	−0.020860007	0.194441609	93.11	1.82236
14	NQA-NAMIBIA	12	0.352181	0.418734	−0.189218	−0.134063327	0.290036023	74.21	1.65979

*He*—expected heterozygosity; *Ho*—observed heterozygosity; *F*—inbreeding coefficient; *F_IS_*—the inbreeding coefficient within populations; mean *F_ST_*—mean pairwise genetic differentiation among populations; *Pn*—proportion of polymorphic SNPs; *Ar*—allelic richness. ED-KAZ—Edilbay breed from Kazakhstan; KFTCW-KAZ—Kazakh fat-tailed coarse-wooled breed from Kazakhstan; GISS-KAZ—Gissar breed from Kazakhstan; TON-MONGOL—Tong breed from Mongolia; TIBQ-CHINA—Tibetan breed from China; KCW-KRG—Kyrgyz coarse-wool breed from Kyrgyzstan; GISS-KRG—Gissar breed from Kyrgyzstan; KRKS-SW-ASIA—Karakas breed from South-West Asia; QEZ-SW-ASIA—Qezel breed from South-West Asia; SOP-ITALY—Sopravissana breed from Italy; OUE-FRANCE—Ouessant breed from France; BBB-USA—Barbados Black Belly breed from Barbados and US; RMA-KENYA—Red Maasai breed from Kenya; NQA-NAMIBIA—Namaqua Afrikaner breed from Namibia.

## Data Availability

The original contributions presented in this study are included in the article. Further inquiries can be directed to the corresponding author.

## Supplementary Materials

The following supporting information can be downloaded at: https://www.mdpi.com/article/10.3390/biology14111478/s1, Figure S1: Admixture analysis of the studied domestic sheep populations: (A) worldwide domestic breeds (optimal *K* = 8). (B) domestic sheep breeds and wild sheep populations (optimal *K* = 8). (C) Delta K graph obtained with the CLUMPAK software; Figure S2: LD Decay across the studied sheep populations including Asian and non-Asian domestic breeds and selected wild sheep populations; Table S1: Worldwide domestic sheep breeds and wild sheep populations included in the study (references [59,60,61,62] are cited in Supplementary Materials). Table S2: Summary statistics of linkage disequilibrium (*r*^2^) across sheep populations. Table S3: Summary of estimated effective population size (Ne) across sheep populations.

## Abbreviations

The following abbreviations are used in this manuscript:

SNP	Single Nucleotide Polymorphism
RSE	Republican State Enterprise
EDTA	Ethylenediaminetetraacetic acid
PCA	Principal Component Analysis
LD	Linkage Disequilibrium
