# Supplementary material for: Genome-Wide SNP Analysis Reveals the Unique Genetic Diversity Represented by Fat-Tailed Coarse-Wooled Sheep Breeds of Kazakhstan
Source: Biology (Basel). 2025 Oct 23;14(11):1478. doi: 10.3390/biology14111478 (PMC12650365; doi:10.3390/biology14111478)
Supplement: Supplementary file 1 [file biology-14-01478-s001.zip › biology-3888189-supplementary/Supplementary_Figures_Dossybayev et.al.pdf]

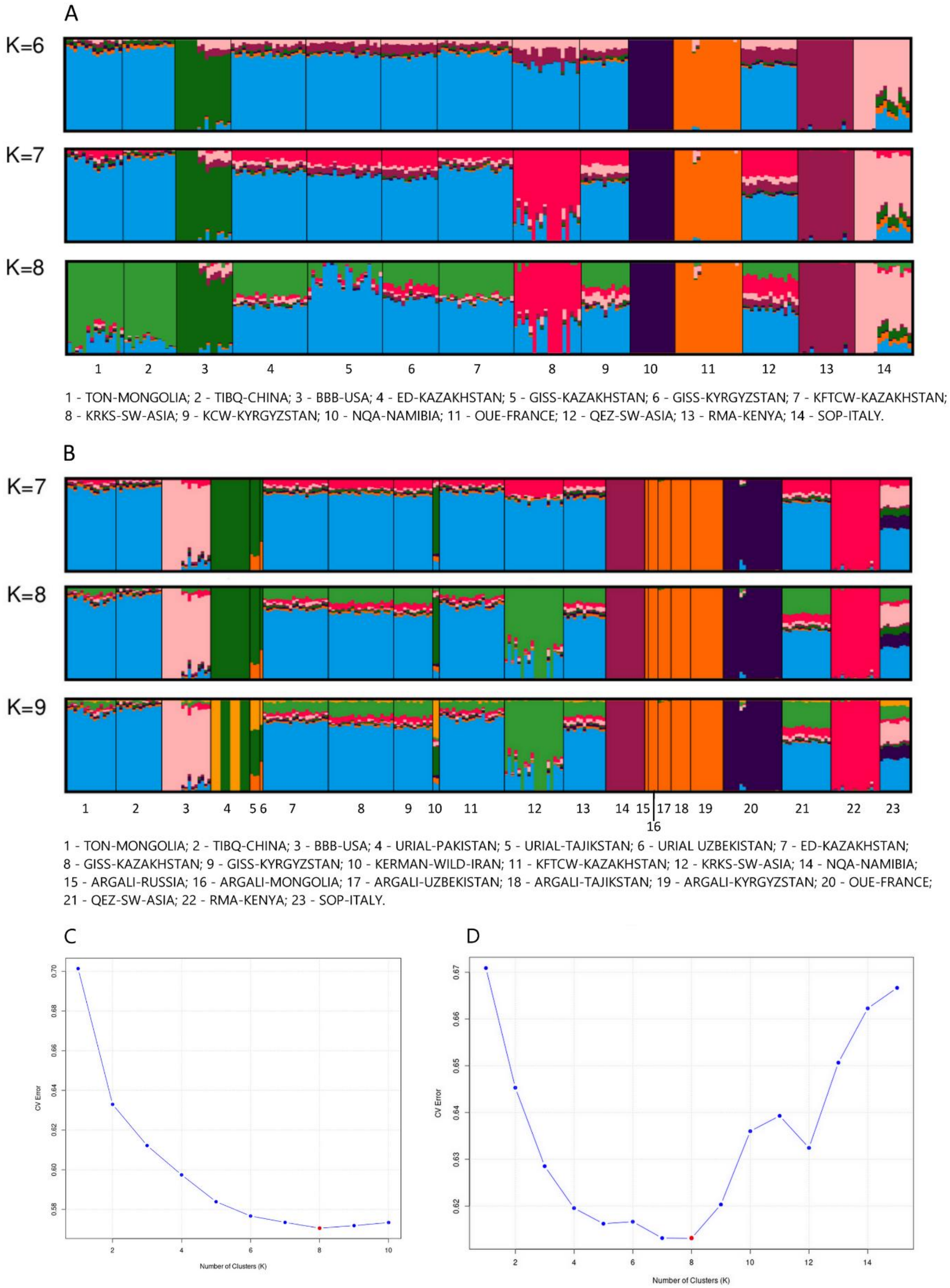

Figure S1. Admixture analysis of the studied domestic sheep populations: (A) worldwide domestic breeds (optimal K=8). (B) domestic sheep breeds and wild sheep populations (optimal K=8). (C) Delta K graph obtained with the CLUMPAK software

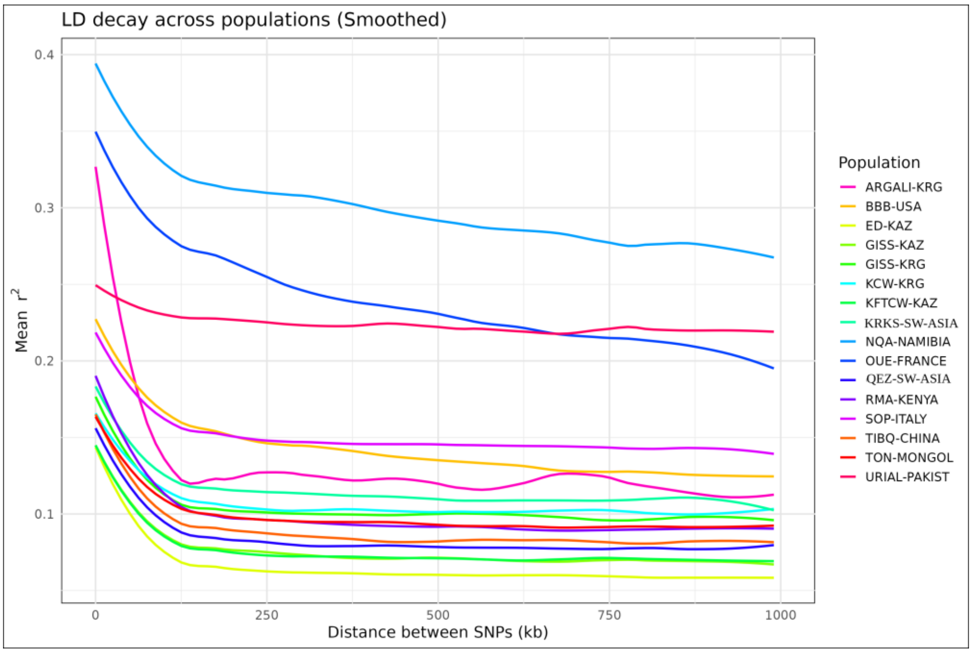

Figure S2. LD Decay across the studied sheep populations including Asian and non-Asian domestic breeds and selected wild sheep populations
