# Supplementary material for: Genome-Wide SNP Analysis Reveals the Unique Genetic Diversity Represented by Fat-Tailed Coarse-Wooled Sheep Breeds of Kazakhstan
Source: Biology (Basel). 2025 Oct 23;14(11):1478. doi: 10.3390/biology14111478 (PMC12650365; doi:10.3390/biology14111478)
Supplement: Supplementary file 1 [file biology-14-01478-s001.zip › biology-3888189-supplementary/Supplementary_Tables_Dossybayev et.al.pdf]

Table S1. Worldwide domestic sheep breeds and wild sheep populations included in the study

№	Sheep breed	Breed characteristics	Number of animals	Country of origin	References
**Domestic sheep breeds**					
1	Tong sheep (TON-MONGOL)	long fat-tailed	15	Mongolia	Yuan, Z. et al., 2016
2	Tibetan sheep (TIBQ-CHINA)	thin-tailed	14	China, Qinghai	Yuan, Z. et al., 2016
3	Kyrgyz coarse-wool sheep (KCW-KRG)	fat-rumped, coarse wool	13	Kyrgyzstan	Deniskova et al., 2019
4	Kyrgyz gissar (GISS-KRG)	fat-tailed	15	Kyrgyzstan	Deniskova et al., 2019
5	Karakas sheep (KRKS-SW-ASIA)	fat-tailed	18	South-West Asia*	Kijas et al., 2012
6	Qezel sheep (QEZ-SW-ASIA)	fat-tailed, coarse wool	15	South-West Asia*	Kijas et al., 2012
7	Sopravissana (SOP-ITALY)	thin-tailed, fine wool	15	Italy	Ciani et al., 2015
8	Ouessant (OUE-FRANCE)	thin-tailed, coarse to medium wool	18	France	Rochus et al, 2018
9	Barbados Black Belly (BBB-USA)	thin-tailed	15	Caribbean (Barbados), also U.S.	Kijas et al, 2012
10	Red Maasai (RMA-KENYA)	fat-tailed	15	Kenya, Tanzania	Kijas et al, 2012
11	Namaqua Afrikaner (NQA-NAMIBIA)	fat-tailed	12	South Africa, Namibia	Kijas et al, 2012
Overall domestic sheep			165		
**Wild sheep**					
12	Urial (URIAL-PAKIST)	thin-tailed, coarse wool	12	Pakistan	Dotsev et.al., 2023
13	Urial (URIAL-TAJIK)	thin-tailed, coarse wool	3	Tajikistan	Dotsev et.al., 2025
14	Urial (URIAL-UZBEK)	thin-tailed, coarse wool	1	Uzbekistan	Dotsev et.al., 2025
15	Kerman or Laristan sheep (KERMAN_WILD-IRAN)	thin-tailed	2	Iran	Dotsev et.al., 2025
16	Argali (ARGALI-RUSSIA)	thin-tailed, coarse wool	1	Russia	Dotsev et.al., 2023
17	Argali (ARGALI-MONGOL)	thin-tailed, coarse wool	3	Mongolia	Dotsev et.al., 2023
18	Argali (ARGALI-UZBEK)	thin-tailed, coarse wool	4	Uzbekistan	Dotsev et.al., 2023
19	Argali (ARGALI-TAJIK)	thin-tailed, coarse wool	6	Tajikistan	Dotsev et.al., 2023
20	Argali (ARGALI-KRG)	thin-tailed, coarse wool	10	Kyrgyzstan	Dotsev et.al., 2023
Overall wild sheep			42		

*South-West Asia (SW-Asia) refers to present day Turkey and Iran, also sometimes referred to as the Middle East.

Table S2. Summary statistics of linkage disequilibrium (r^2) across sheep populations

Population	Mean_R2	Median_R2	SD_R2	Max_R2	Pairs_N
RMA-KENYA	0.0969	0.0488	0.1257	1.0	249030
GISS-KAZ	0.0746	0.0379	0.0969	1.0	277652
KERMAN_WILD-IRAN	1.0	1.0	0.0	1.0	44698
ARGALI-TAJIK	0.2163	0.1111	0.2343	1.0	9557
KCW-KRG	0.1052	0.0545	0.1283	1.0	273326
OUE-FRANCE	0.2375	0.1176	0.2762	1.0	185509
KRKS-TURKEY	0.114	0.0621	0.1383	1.0	265238
TON-MONGOL	0.0968	0.051	0.1207	1.0	268995
BBB-USA	0.1407	0.0767	0.1695	1.0	249661
QEZ-IRAN	0.0825	0.0428	0.1054	1.0	275753
URIAL-PAKIST	0.2235	0.1488	0.2354	1.0	131048
ARGALI-MONGOL	0.522	0.25	0.3609	1.0	3173
ARGALI-UZBEK	0.4598	0.3333	0.3546	1.0	10433
NQA-NAMIBIA	0.2974	0.1818	0.2984	1.0	160128
GISS-KRG	0.1024	0.0556	0.1265	1.0	273306
KFTCW-KAZ	0.0746	0.0382	0.0968	1.0	278732
SOP-ITALY	0.1488	0.0833	0.1709	1.0	248421
URIAL-TAJIK	0.5601	0.25	0.363	1.0	60730
ED-KAZ	0.064	0.0318	0.0847	1.0	280715
TIBQ-CHINA	0.0874	0.0455	0.1123	1.0	265857
ARGALI-KRG	0.1269	0.0625	0.1735	1.0	14896

Mean, median, standard deviation (SD), and maximum values of pairwise linkage disequilibrium (r^2). The number of SNP pairs used in each population is indicated as *Pairs_N*. ED-KAZ – Edilbay breed from Kazakhstan; KFTCW-KAZ – Kazakh fat-tailed coarse-wooled breed from Kazakhstan; GISS-KAZ – Gissar breed from Kazakhstan; TON-MONGOL – Tong breed from Mongolia; TIBQ-CHINA – Tibetan breed from China; KCW-KRG – Kyrgyz coarse-wool breed from Kyrgyzstan; GISS-KRG – Gissar breed from Kyrgyzstan; KRKS-SW-ASIA – Karakas breed from South-West Asia; QEZ-SW-ASIA – Qezel breed from South-West Asia; SOP-ITALY – Sopravissana breed from Italy; OUE-FRANCE – Ouessant breed from France; BBB-USA – Barbados Black Belly breed from Barbados and US; RMA-KENYA – Red Maasai breed from Kenya; NQA-NAMIBIA – Namaqua Afrikaner breed from Namibia.

Table S3. Summary of estimated effective population size (Ne) across sheep populations

Population	Mean_Ne	Median_Ne	Min_Ne	Max_Ne	Recent_Ne
ARGALI-UZBEK	52.87	37.0	15	146	15.0
GISS-KRG	888.64	422.0	61	2910	61.0
OUE-FRANCE	306.25	145.5	42	1189	42.0
GISS-KAZ	1339.04	693.0	95	4211	95.0
ED-KAZ	1528.12	830.5	111	4294	111.0
SOP-ITALY	608.16	276.0	40	2139	40.0
QEZ-SW-ASIA	1195.19	619.5	78	3516	78.0
KCW-KRG	887.48	420.0	59	3049	59.0
ARGALI-TAJIK	104.4	76.0	28	270	28.0
KRKS-SW-ASIA	797.32	380.0	57	2739	57.0
RMA-KENYA	895.48	472.0	68	2677	68.0
NQA-NAMIBIA	262.17	117.5	22	938	22.0
URIAL-PAKIST	352.38	153.0	23	1313	23.0
ARGALI-KRG	326.78	201.5	54	1055	54.0
TON-MONGOL	959.2	464.0	69	3389	69.0
TIBQ-CHINA	1055.84	541.0	75	3453	75.0
KFTCW-KAZ	1370.58	693.0	92	4547	92.0
BBB-USA	591.64	296.0	56	2047	56.0
URIAL-TAJIK	112.95	56.0	12	449	12.0

Mean, median, minimum, maximum, and recent (most contemporary) estimated effective population sizes (Ne). *Recent_Ne* corresponds to the Ne value at the smallest generation ago (GenAgo), representing the most recent population size estimate. ED-KAZ – Edilbay breed from Kazakhstan; KFTCW-KAZ – Kazakh fat-tailed coarse-wooled breed from Kazakhstan; GISS-KAZ – Gissar breed from Kazakhstan; TON-MONGOL – Tong breed from Mongolia; TIBQ-CHINA – Tibetan breed from China; KCW-KRG – Kyrgyz coarse-wool breed from Kyrgyzstan; GISS-KRG – Gissar breed from Kyrgyzstan; KRKS-SW-ASIA – Karakas breed from South-West Asia; QEZ-SW-ASIA – Qezel breed from South-West Asia; SOP-ITALY – Sopravissana breed from Italy; OUE-FRANCE – Ouessant breed from France; BBB-USA – Barbados Black Belly breed from Barbados and US; RMA-KENYA – Red Maasai breed from Kenya; NQA-NAMIBIA – Namaqua Afrikaner breed from Namibia.
